# Off-pump coronary bypass grafting for kawasaki disease

**DOI:** 10.4103/0974-2069.74058

**Published:** 2010

**Authors:** Sudeep Verma, Charumathi Dasarathan, R Premsekar, Prashanth Vaijyanath

**Affiliations:** Department of Pediatric Cardiology and Cardiovascular Thoracic Surgery, International Institute of Cardiovascular and Thoracic Surgery, Frontier Life Line Hospital, Dr. K. M Cherian Heart Foundation, Chennai, Tamil Nadu, India

**Keywords:** Kawasaki disease, off-pump coronary artery bypass graft, right coronary artery

## Abstract

A 6-year-old girl, a known case of Kawasaki disease, presented with acute thrombotic occlusion of right coronary artery (RCA) with symptoms of acute angina and myocardial dysfunction. She underwent beating heart off-pump coronary artery bypass graft (CABG) surgery with right internal mammary artery (RIMA) grafted to distal RCA. Follow-up computed tomography angiogram revealed well-flowing RIMA with no obstruction or kink. This case highlights the importance of CABG as a safe and life-saving procedure in expert hands, even for children in emergent conditions.

## INTRODUCTION

Kawasaki disease (KD) is perhaps the most common vasculitic disorder in children, with unknown etiology. Coronary aneurysms develop in 15–25%, usually within 6–8 weeks after the onset of illness, and are responsible for myocardial infarction (<5%) and mortality (1–5%). Angiographic resolution of aneurysms 1–2 years after the illness occurs in 50–67% of the patients.[1] Despite the high incidence of KD all over the world, the number of cases reported from India remain meagre.[2,3]

## CASE REPORT

**Figure 1:**
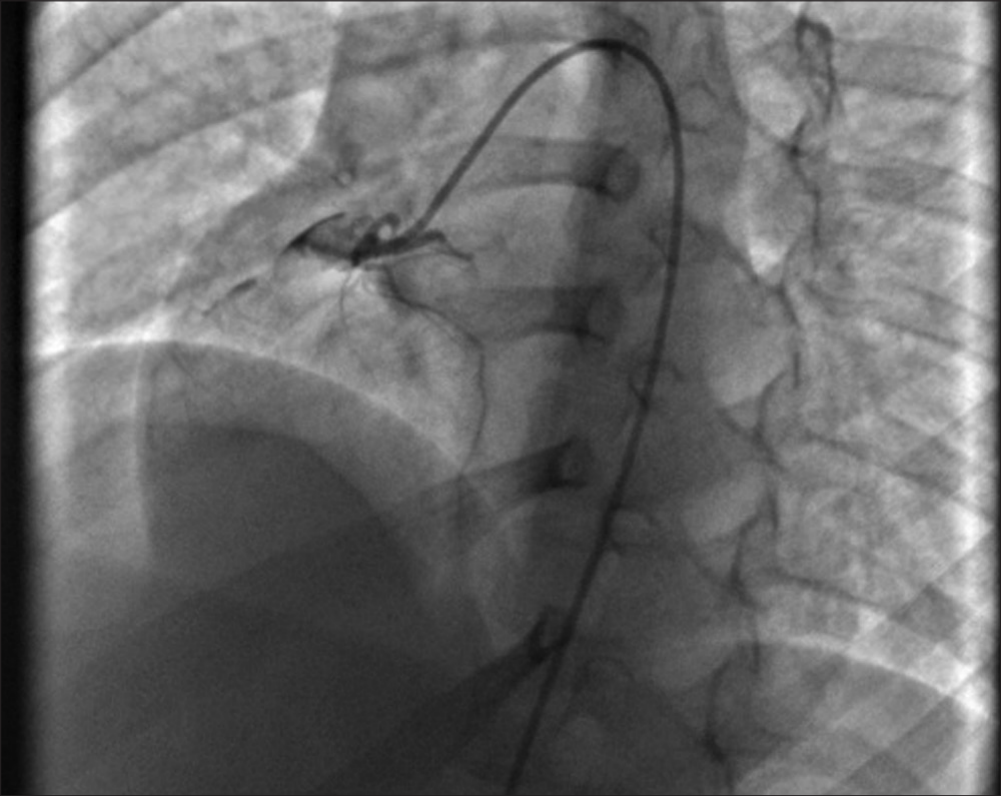
Coronary angiogram in left anterior oblique (LAO) 40° view showing total occlusion of RCA with absent distal flow

**Figure 2:**
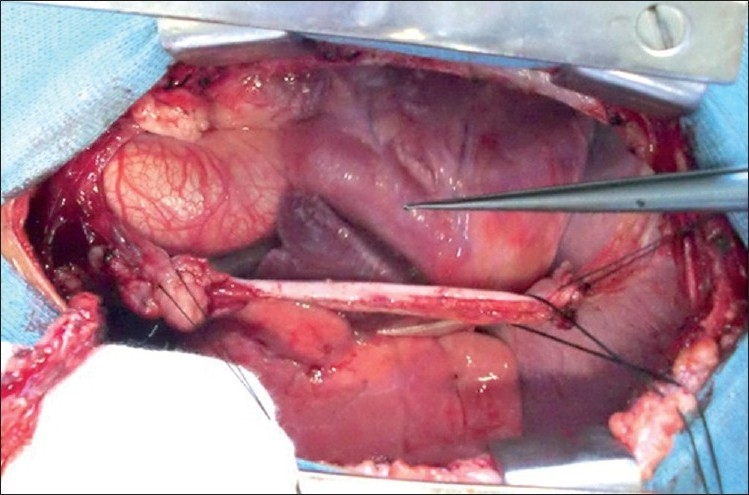
Surgical picture of aneurysmally dilated and thrombosed RCA extending upto midsegment

**Figure 3:**
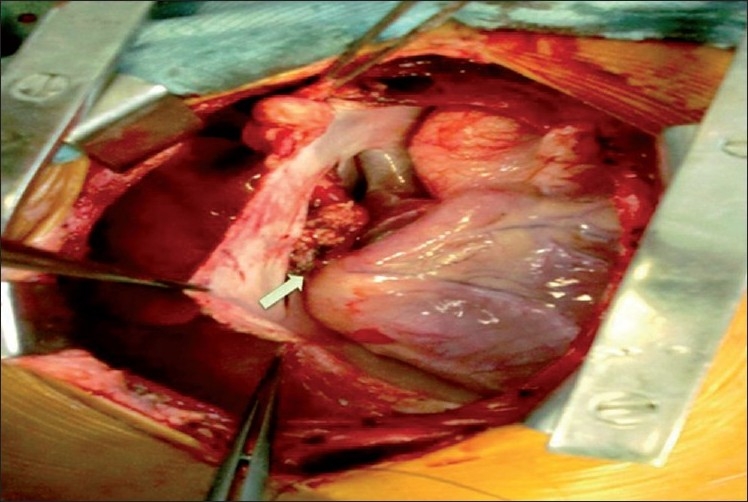
Surgical picture of RIMA grafted to distal RCA

**Figure 4:**
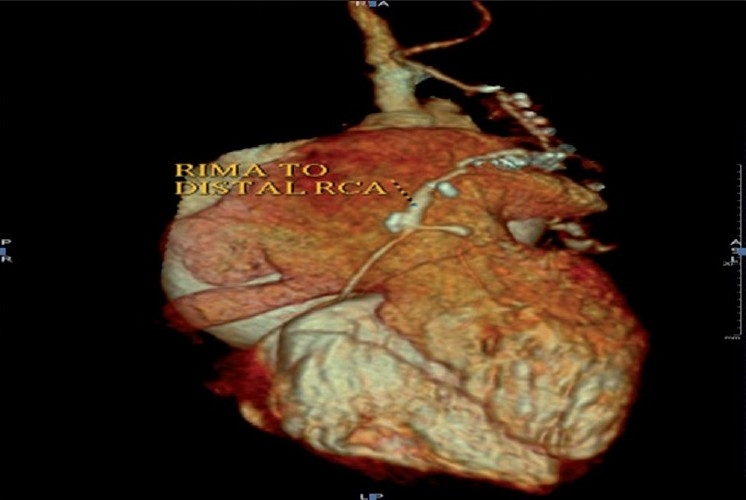
Postoperative CT angiogram picture showing the patent RIMA graft with interposed saphenous venous graft

A 6-year-old girl child was admitted with complaints of severe pain over the shoulder and jaw, associated with excessive sweating and dyspnea, 12 hours preceding admission. She was diagnosed with KD 6 months previously and had received two doses of intravenous immunoglobulin at that time and was on aspirin and clopidogrel, with history of reactivation of KD along with Reye like illness 3 months after the initial episode. At admission, electrocardiogram showed ischemic changes in lead II, III and avF. Echocardiography revealed regional wall motion abnormality with left ventricular dysfunction. Laboratory investigations showed increased levels of serum myocardial enzymes. Coronary angiography revealed total thrombotic occlusion of an aneurysmally dilated proximal RCA [Figure 1] with retrograde filling of posterior descending artery from a normal left coronary artery and a good sized RIMA. Operative findings included a 15-mm thrombotic RCA aneurysm extending up to the mid-RCA [Figure 2]. She underwent off-pump beating heart revascularization procedure with RIMA grafted to distal RCA [Figure 3] with interposition saphenous graft, as RIMA was short for the anastomosis. Since KD is a medium-sized vessel vasculitis, a saphenous interposition venous graft was used. Also, harvesting a radial graft is risky with more chances of complications in children. The postoperative stay was uneventful and the child was discharged 7 days after CABG with fully recovered ventricular function. Follow-up CT angiogram, 2 weeks after the discharge, revealed a well-flowing RIMA with distal RCA anastomosis with no evidence of any obstruction or kink [Figure 4].

## DISCUSSION

Surgical revascularization for coronary artery disease secondary to KD is relatively uncommon. Aneurysms tends to develop most frequently in the left main coronary artery (LMCA) followed by left anterior descending artery (LAD) and RCA.[4] The left circumflex artery (LCC) is least often involved.[4] Significantly higher temperature on days 9–12 and longer duration of fever (>14 days) appear to be the risk factors for developing coronary aneurysms. Generalized microvasculitis occurs throughout the body in the first 10 days of the disease. Myocardium is affected mainly in the first 3–4 weeks, with valvulitis involving mitral and aortic valves. Inflammation persists in the coronary arteries, characterized by edema, mononuclear cell infiltration and progressive fibrosis with internal elastic lamina destruction and aneurysm formation.

These changes are more pronounced in the proximal segments and branching points of coronary arteries, suggesting the role of hemodynamic stress.[5] The resolution appears to be more likely with a smaller aneurysm, age at onset younger than 1 year, fusiform rather than saccular aneurysms, and aneurysms located at a distal coronary segment.[6] KD recurs in 2–3% of patients who recover completely from the original episode, with increased risk of coronary complications. Myocardial ischemia or infarction occurring in the later periods may be due to acute thrombosis or progressive coronary artery stenosis. Giant aneurysms (>8 mm internal diameter) present a disproportionately higher risk of myocardial infarction as compared to aneurysms of smaller dimensions.[7] Rarely, aneurysms may rupture and cause sudden death mostly associated with steroid treatment in acute phase.[8] CABG is indicated when one of the major arteries gets occluded with thrombus, with evidence of ischemia. The use of internal thoracic arteries resulted in 100% 1 year graft patency rate.[9,10] In order to avoid the acute complications like rupture, myocardial infarction and death, coronary artery aneurysm should be managed aggressively. Surgical off-pump revascularization using internal mammary artery in expert hands is a safe and an effective treatment modality in young patients with good long-term graft patency rate. When additional grafts are required, there is no evidence to suggest that either the radial artery or saphenous vein is superior.[11] Although very less data are available for the off-pump CABG (OPCAB) in children, anecdotal reports show encouraging results.[12,13] The decision between OPCAB and conventional CABG has to weigh several factors, including the likely risks and benefits of the two approaches for the particular patient, the experience of the surgeon, the complexity of the coronary disease, and the required coronary revascularization.[14] Less blood loss and need for transfusion, less myocardial enzyme release, less early neurocognitive dysfunction, and less renal insufficiency are the probable benefits with OPCAB, but only fewer grafts tend to be performed with OPCAB than with standard CABG. Length of hospital stay, mortality rate, and long-term neurological function and cardiac outcome appear to be similar in the two groups.[15]

Coronary bypass operation is a safe and reliable surgical modality for coronary artery sequelae in children with KD and it should be considered as a possible treatment modality for coronary revascularization even in young children. This procedure remains a technical challenge and requires careful follow up.
